# Convexity subarachnoid haemorrhage has a high risk of intracerebral haemorrhage in suspected cerebral amyloid angiopathy

**DOI:** 10.1007/s00415-017-8398-y

**Published:** 2017-02-02

**Authors:** D. Wilson, I. C. Hostettler, G. Ambler, G. Banerjee, H. R. Jäger, D. J. Werring

**Affiliations:** 1 Stroke Research Centre, UCL Institute of Neurology, University College London, Russell Square House, 10-12 Russell Square, London, WC1B 5EH UK; 2 Department of Statistical Science, UCL, London, WC1E 6BT UK; 3 Neuroradiological Academic Unit, Department of Brain Repair and Rehabilitation, Institute of Neurology, University College London, London, UK

**Keywords:** Non-traumatic convexity/convexial/cortical subarachnoid haemorrhage, Intracerebral haemorrhage, Cerebral amyloid angiopathy, Superficial siderosis, Stroke

## Abstract

The risk of future symptomatic intracerebral haemorrhage (sICH) remains uncertain in patients with acute convexity subarachnoid haemorrhage (cSAH) associated with suspected cerebral amyloid angiopathy (CAA). We assessed the risk of future sICH in patients presenting to our comprehensive stroke service with acute non-traumatic cSAH due to suspected CAA, between 2011 and 2016. We conducted a systematic search and pooled analysis including our cohort and other published studies including similar cohorts. Our hospital cohort included 20 patients (mean age 69 years; 60% male); 12 (60%) had probable CAA, and 6 (30%) had possible CAA according to the modified Boston criteria; two did not meet CAA criteria because of age <55 years, but were judged likely to be due to CAA. Fourteen patients (70%) had cortical superficial siderosis; 12 (60%) had cerebral microbleeds. Over a mean follow-up period of 19 months, 2 patients (9%) suffered sICH, both with probable CAA (annual sICH risk for probable CAA 8%). In a pooled analysis including our cohort and eight other studies (*n* = 172), the overall sICH rate per patient-year was 16% (95% CI 11–24%). In those with probable CAA (*n* = 104), the sICH rate per patient-year was 19% (95% CI 13–27%), compared to 7% (95% CI 3–15%) for those without probable CAA (*n* = 72). Patients with acute cSAH associated with suspected CAA are at high risk of future sICH (16% per patient-year); probable CAA might carry the highest risk.

## Introduction

Non-traumatic acute convexity subarachnoid haemorrhage (cSAH)—also known as acute cortical subarachnoid haemorrahge—is confined to the subarachnoid space over the cortical hemispheric convexities of the brain, and does not extend into the parenchyma, sylvian fissures, ventricles, or basal cisterns [1]. The aetiology and symptoms of cSAH are diverse, but have been classified by age of presentation [1–7]; in younger patients (usually <60 years), cSAH often occurs in association with reversible cerebral vasoconstriction syndrome (RCVS), with recurrent thunderclap headache as the predominant presenting symptom [8]. RCVS generally has a favourable outcome with very low recurrence risk, although in the acute phase both ischaemic stroke and intracerebral haemorrhage (ICH) can occur in a minority of patients [9]. By contrast, in older patients, cSAH is associated with cerebral amyloid angiopathy (CAA) [10], often presenting with transient focal neurological episodes (TFNE), often consisting of unilateral spreading sensory or motor symptoms. In CAA there is a substantial risk of recurrent symptomatic ICH (sICH) after presentation with ICH [11], but knowledge about ICH risk following cSAH in patients with suspected CAA is limited, comprising only small cohort studies which individually contain very few clinical outcome events. A pooled meta-analysis of event rates from multiple prospective cohort studies allows a more precise estimate of sICH risk than is available from any individual study. This information is important in guiding clinical decision making with regard to prognosis and antithrombotic treatment, a common management dilemma in older patients at risk of vaso-occlusive events, for example those with atrial fibrillation or ischaemic heart disease.

We therefore investigated the risk of sICH following cSAH in: (1) a cohort of patients from our comprehensive stroke service presenting with acute symptomatic cSAH due to suspected CAA and (2) a pooled analysis which included our cohort and all other available published data on cSAH in suspected CAA cohorts.

## Materials and methods

### Data collection

#### Hospital based cohort study

We retrospectively searched prospective radiological and clinical databases of patients who were assessed in our specialist comprehensive stroke service (University College Hospital and the National Hospital for Neurology and Neurosurgery, Queen Square) between 2011–2016 and diagnosed with suspected CAA; our search terms were “convexity subarachnoid haemorrhage” and “cortical subarachnoid haemorrhage”. Our inclusion criteria were: acute symptomatic cSAH visualised on CT or MRI, with exclusion of non-CAA causes of cSAH (including RCVS, cerebral venous sinus thrombosis, aneurysmal SAH or traumatic SAH), and the availability of follow-up sICH data. Information on patient demographics, risk factor profile, history of previous ICH, and follow up data was obtained from electronic medical records which clearly confirmed the absence or presence of new symptoms or signs of stroke. Our outcome of interest was the presence of sICH confirmed on neuroimaging (CT or MRI) during the follow-up period. Markers of cerebral small vessel disease were rated by a postgraduate clinical neurology trainee PhD student (DW) trained by a consultant vascular neuro-radiologist with reproducibility against other observers confirmed in previous projects (with similar cohorts and imaging acquisition), consistently obtaining Kappa values between 0.6 and 0.8 for cerebral microbleeds (CMBs), cortical superficial siderosis, MRI-visible enlarged perivascular spaces (EPVS) and white matter hyperintensities. Cerebral microbleeds were rated on T2*-weighted or susceptibility-weighted imaging using the MARS scale [12], white matter hyperintensities were rated on FLAIR sequences using the simplified Fazekas scale [13], and EPVS were rated on T2-weighted sequences using a validated four point rating scale [14]. Cortical superficial siderosis was rated using as focal or disseminated using a previously developed scale [15] (siderosis was not rated if it corresponded to the acute cSAH) according to standardised criteria [16]. Brain imaging was also reviewed to assess whether or not each patient met the modified Boston diagnostic criteria for probable or possible CAA [15].

#### Systematic review and pooled meta-analysis

Two authors (DW and ICH) searched Medline for cohort studies of cSAH using key words “convex* adj4 subarachnoid” OR cortical adj4 subarachnoid OR sulc* adj4 subarachnoid on the 20/05/2016, in addition to the reference lists. We did not limit the search terms to any language or dates. Only studies including participants with suspected CAA with no other obvious cause for their cSAH, and at least 6 months of follow-up data, were included. The two authors reviewed all papers and independently extracted the following information: population from which patients were drawn, number of patients, mean follow-up time, and outcome events (sICH and death). In two studies, follow up data were given as median values; these were converted to mean values using a validated method [16–18]. Our cohort study is reported according to the STROBE guidelines, and the pooled-analysis was conducted according to PRISMA guidelines [19].

### Statistical analysis

We calculated the pooled risk of sICH for patients with cSAH and suspected CAA using a mixed effects Poisson model to account for studies with no ICH outcomes. We then calculated the risks of sICH in patients who fulfilled the Boston criteria for probable CAA, and for those who did not fulfil these criteria. Meta-analysis was used to create forest plots for patients fulfilling probable CAA and for those who did not fulfil this criteria. Rates were calculated per patient year and exact Poisson 95% confidence intervals were calculated for each study. Statistical analysis was performed using STATA 13 (StataCorp. 2011; Stata Statistical Software: Release 13; College Station, TX: StataCorp LP).

### Ethical approval

Data collection was approved as a Service Evaluation in the Comprehensive Stroke Service, University College London Hospitals NHS Foundation Trust. The study was also approved by an NHS Health Research Authority Research Ethics Committee (reference: 15/LO/1443).

## Results

### Hospital-based cohort study

We identified 27 patients with cSAH between January 2011 and February 2016. After excluding those diagnosed with causes other than suspected CAA (RCVS or aneurysmal cSAH) and cases where cSAH was diagnosed incidentally without clinical suspicion of CAA, we included 20 patients. The median age was 69 years and 60% were male. The demographic and clinical characteristics of the cohort are shown in Table 1. Of the 20 patients, 12 had probable CAA according to the modified Boston criteria [15], while 6 had possible CAA. Two patients, aged 50 and 53, were still considered suspected to have CAA despite being less than 55 years old.

**Table 1:** Characteristics of hospital-based cohort of patients with suspected CAA

Patient	Age	Sex	Location of cSAH	Symptoms at presentation	Positive or negative symptoms	Spreading symptoms?	CMB number (all lobar)	Siderosis	CAA^a^	WMH^b^	EPVS score^c^	Previous ICH	ICH at follow up
1	59	F	R fronto-parietal	Sensory	Positive	NA	0	Focal	Possible	0	3	0	0
2	84	F	L fronto-parietal	Sensory	Positive	Yes	3	Diss	Probable	2	2	0	0
3	82	M	R fronto-parietal	Sensory	Positive	Yes	6	Diss	Probable	1	2	0	0
4	75	F	L fronto-parietal	Motor and sensory	Negative	Yes	0	Diss	Possible	0	3	0	0
5	63	F	L frontal	Speech and sensory	Negative	No	3	Focal	Probable	2	3	0	0
6	78	F	R parietal	Motor and sensory	Both	No	4	Diss	Probable	1	2	0	1
7	67	M	L frontal	Motor and speech	Both	Yes	0	Diss	Probable	2	2	Lobar	0
8	82	M	L frontal	Sensory	Positive	Yes	1	Diss	Probable	0	3	0	1
9	70	M	R fronto-parietal	Sensory	Positive	Yes	0	Focal	Possible	0	2	0	0
10	76	F	L fronto-parietal	Swallow	Negative	No	3	Diss	Probable	0	4	0	0
11	64	M	R fronto-parietal	Sensory and speech	Both	No	2	Diss	Probable	2	3	0	0
12	62	M	R frontal	Motor	Negative	Yes	5	Focal	Probable	2	2	Lobar	0
13	76	M	R fronto-parietal	Motor	Negative	No	3	Diss	Probable	2	1	0	0
14	76	M	R frontal	Sensory	Positive	Yes	5	Diss	Probable	0	2	0	0
15	73	M	R fronto-parietal	Sensory	Negative	No	0	Diss	Possible	3	3	0	0
16	50	F	Bifrontal	Sensory	Positive	No	4	Diss	Suspected^d^	0	3	0	0
17	72	F	L fronto-parietal	Sensory	Positive	Yes	0	Focal	Possible	2	2	0	0
18	81	M	L frontal	Sensory	Positive	Yes	1	Diss	Probable	0	4	0	0
19	63	F	L frontal	Motor	Negative	No	0	Focal	Possible	0	1	0	0
20	53	M	Bi fronto-parietal	Headache and LOC	Negative	No	0	Absent	Suspected^d^	0	1	0	0

*cSAH* convexity subarachnoid haemorrhage, *CMB* cerebral microbleed, *Diss* disseminated, *EPVS* enlarged perivascular spaces

^a^According to the modified Boston criteria

^b^WMH—white matter hyperintensities (leukoaraiosis) measured by simplified Fazekas criteria

^c^EPVS measured using a validated 4 point scale [14]

^d^Age <55

Cerebral microbleeds (CMBs) were present in 12 of 20 (60%) of patients, with a median of 1 (IQR 0–4). All CMBs were lobar. Cortical superficial siderosis (cSS) was present in 19 of 20 (95%) of patients; this was outside the region of their acute cSAH in 15 of 20 patients (75%) and, in these cases, was disseminated (>3 sulci) in 13 of 15 patients (87%). Severe white matter hyperintensities (defined as either simplified Fazekas ≥2 in periventricular or deep white matter) were present in 8 of the 20 patients (40%). MRI-visible perivascular spaces of moderate or severe grade (grades 3 or 4) were present in the centrum semiovale in 9 of 20 patients (45%).

**Fig. 1:**
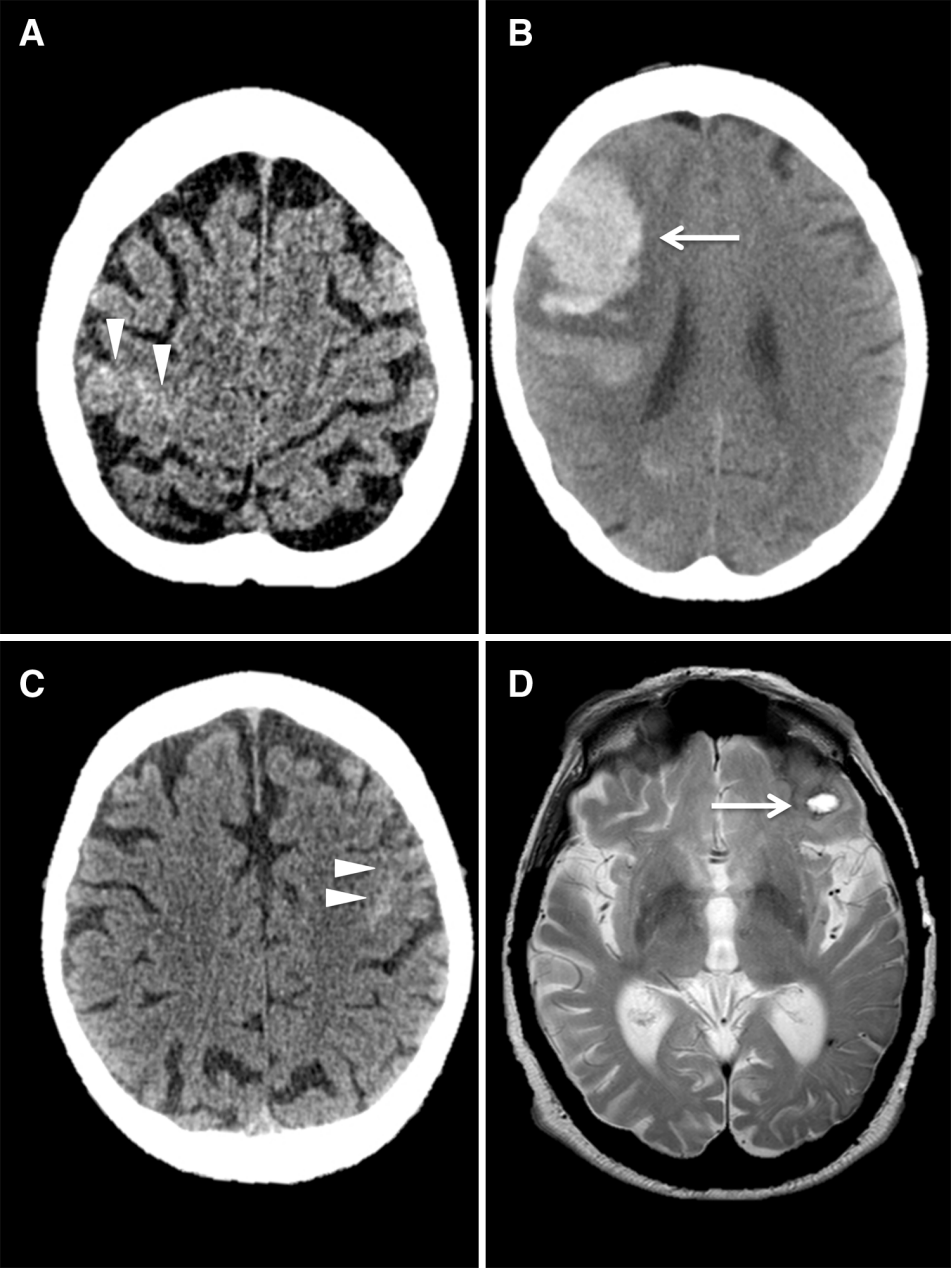
**a** Axial CT scan shows acute cSAH in the right central sulcus (*white arrowheads*); **b** follow-up axial CT shows a subsequent right frontal ICH in the same patient as *panel* (**a**) (*white arrow*); **c** axial CT scan shows acute cSAH in a left frontal lobe sulcus (*white arrowheads*); **d** a follow-up axial T2-weighted MRI shows an acute left frontal ICH in the same patient as *panel* (**c**) (*white arrow*)

All but one of our 20 patients had transient focal neurological episodes (TFNE) as their presenting symptom (one had headache and loss of consciousness); 8 (40%) had only negative symptoms (e.g. weakness, numbness), while 12 (60%) had positive symptoms (e.g. paraesthesias). Three of the 12 patients with positive symptoms also had negative symptoms. Ten patients (50%) had clearly migratory symptoms spreading smoothly from one body part to another (e.g. from the hand, up the arm and into the face). Patients were followed up for a mean of 568 days (IQR 46–683 days). During this period, there were 2 sICH (Fig. 1), and 1 ischaemic stroke. Both of the sICH occurred in patients with ‘probable CAA’ (corresponding to an annual sICH risk of 9% in patients with probable CAA), neither of whom had experienced a previous ICH. There were two deaths in our cohort during follow up (1 from sICH; the other unrelated to sICH).

### Systematic review and pooled analysis

**Fig. 2:**
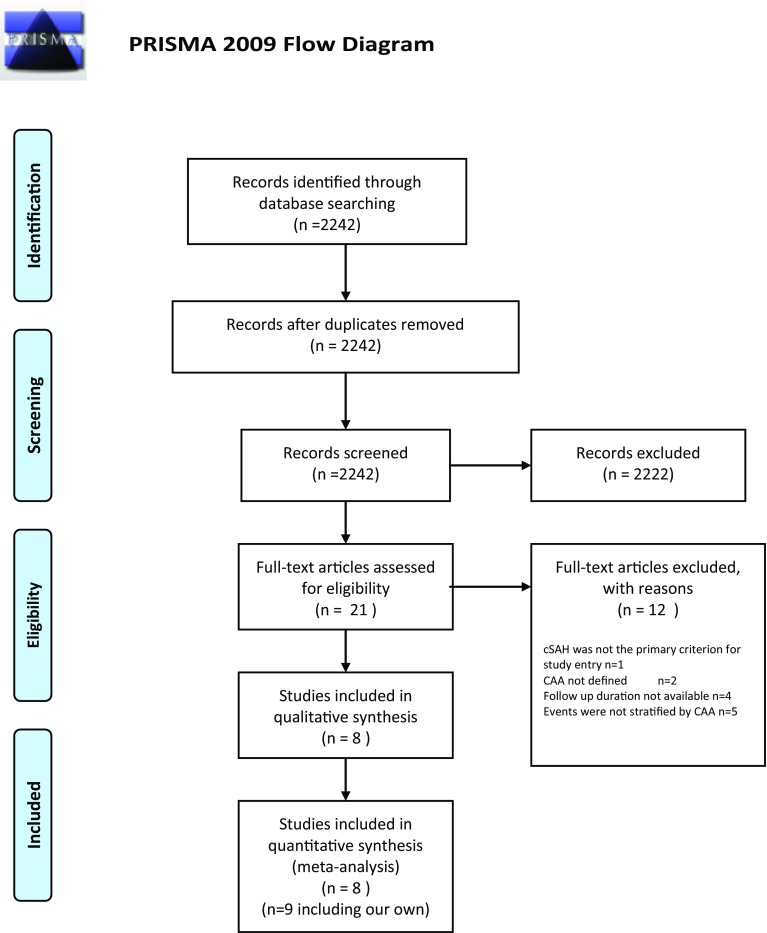
Systematic search flow diagram

Our systematic search terms yielded 2242 papers. After reviewing these abstracts, 20 studies were eligible. Of these, a further 12 were excluded (1 study did not have cSAH as an inclusion criterion; 2 did not document which patients had probable CAA; 4 did not provide average (mean or median) follow-up; and 5 did not specify whether sICH events occurred in those with probable CAA). Thus, eight studies [10, 17, 20–28] of cSAH attributed to suspected CAA were included in the meta-analysis, in addition to our own cohort (see Fig. 2 for a flow chart of study selection). The baseline characteristics of each of the included studies are shown in Table 2. A total of 172 patients were included, 100 fulfilling criteria for probable CAA and 72 not fulfilling criteria for probable CAA.

**Table 2:** Characteristics of studies included in pooled analysis of the rate of symptomatic ICH during follow-up

Study	Patient population	Retrospective/prospective	Total patient No.	Patients with probable CAA	CSS	Mean study follow up (months)	HTN *n* (%) Entire paper	Previous ICH *n* (%) Entire paper	Average age Entire paper	ICH events	ICH events in probable CAA patients
Apoil et al. [20]	Symptomatic cSAH	Retrospective consecutive	17	7	15	28	9 (53)	3 (18)	78	5	5
Beitzke et al. [21]	All cSAH	Retrospective consecutive	38	19	26	24	19 (49)	4 (10)	77 (SD 11)	14	11
Calviere et al. [22]	cSAH fulfilling Boston criteria for CAA	Retrospective consecutive	20	20	NK	30	16 (70)	13 (57)	76 (SD 7)	6	6
Geraldes er al. [23]	All cSAH	Retrospective consecutive	5	0	NK	30	5 (33)	0	60	2	0
Graff-Radford et al. [17]	All cSAH	Retrospective consecutive	37	23	NK	22	NA	1 (1)	64	12	10
Ly et al. [24]	cSAH fulfilling Boston criteria for CAA	Prospective consecutive	7	7	NK	41	NA	NA	74 (SD 10)	3	3
Mas et al. [25]	All cSAH	Retrospective consecutive	16	3	7	12	NA	NA	70	2	0
Raposo et al. [10]	cSAH without obvious cause (RCVS excluded)	Retrospective consecutive	10	8	9	6	9 (90)	1 (10)	74 (SD 9)	1	1
Wilson	All cSAH	Retrospective consecutive	22	13	21	18.7	11	2 (10%)	72.5 (SD 9.42)	2	2

**Fig. 3:**
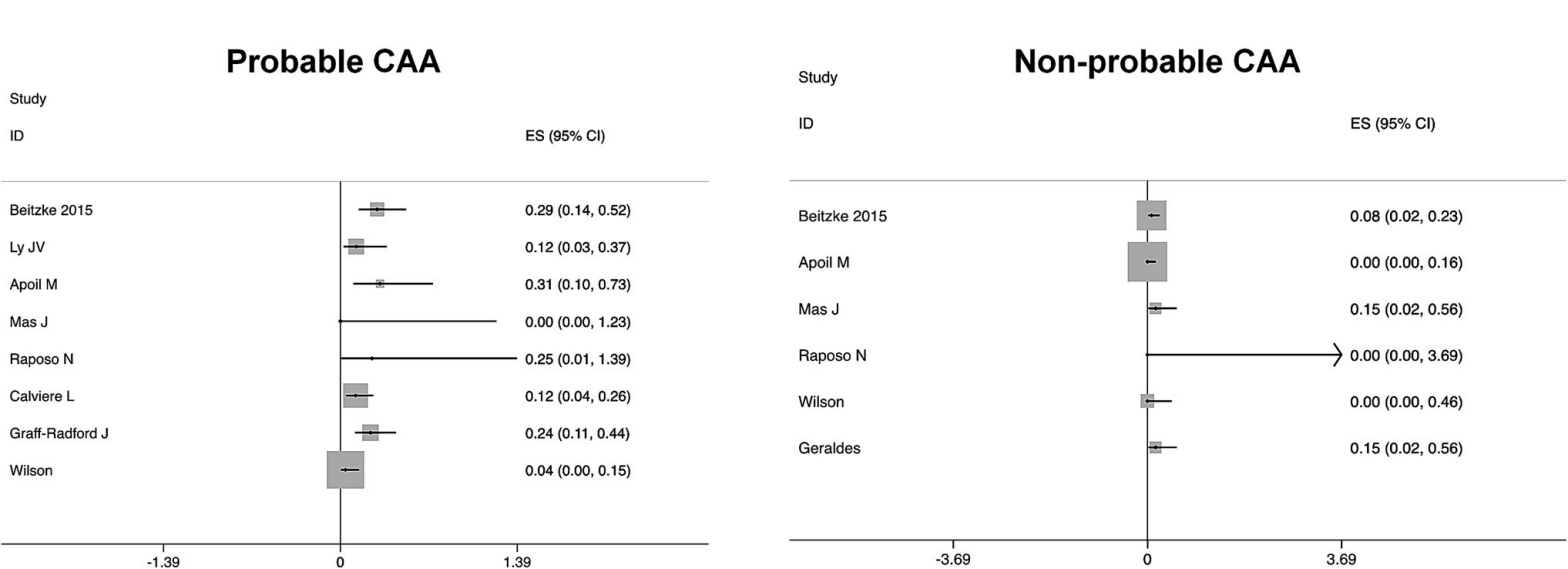
Forest plot showing risk of symptomatic ICH from individual studies in patients presenting with convexity subarachnoid haemorrhage fulfilling and not fulfilling the modified Boston criteria for probable CAA

In a pooled analysis of all 172 patients with cSAH, the absolute sICH event rate per patient-year for patients with suspected CAA was 0.16 (95% CI 0.11–0.24). The pooled absolute sICH event rate per patient-year for those with probable CAA (*n* = 100) was 0.19 (95% CI 0.13–0.27); there was little heterogeneity between studies (*I* ^2^ = 31%) (Fig. 3). For those not fulfilling criteria for probable CAA (*n* = 72) the pooled event rate per patient-year was 0.07 (95% CI 0.03–0.15), with minimal heterogeneity (*I* ^2^ = 0%).

In a sensitivity analysis including a recently published cohort of 27 survivors of lobar ICH attributed to CAA with acute cSAH and available follow up [29] the results remained similar, with a future risk of sICH per patient-year of 0.17 (95% CI 0.12–0.25). This cohort was not included in the main analysis as patients had both lobar ICH and concurrent cSAH at presentation, so are different to those in the other studies we included.

## Discussion

In our hospital cohort study the risk of symptomatic ICH following cSAH in patients with probable CAA was 4% per year; this risk was unrelated to prior ICH, as only 2 of 20 patients (5%) had a history of prior ICH (and they did not have a sICH during follow up). Most previous data suggesting a high future sICH risk in patients with suspected CAA (of around 9–16% per year [11, 30]) are from populations of patients who presented with ICH. In our pooled analysis the annual risk of symptomatic ICH after cSAH due to suspected CAA was similarly high, at 19% per year (95% CI 13–27%) for patients with probable CAA, although lower, at 7% (95% CI 3–15%), for patients who did not meet the modified Boston criteria for probable CAA.

We also found that those with cSAH nearly always had cSS, with a high prevalence of disseminated cSS and severe centrum semiovale MRI-visible perivascular spaces, lobar CMBs and severe WMH (leukoaraiosis). The almost universal presence of cSS (often disseminated) and high risk of ICH following cSAH in our cohort is consistent with evidence that siderosis-predominant CAA is a distinct subtype, with a high risk of intracranial (and convexity subarachnoid) haemorrhage compared to CAA with predominant CMBs or leukoaraiosis [31]. Notably, in our cohort, the prevalence of cSS extending beyond that secondary to the index cSAH was higher than in typical CAA-related ICH populations [32]. A recent study suggested that active leptomeningeal CAA (with contrast enhancement) is the likely cause of CAA-related cSS [27]. Thus, the high prevalence of disseminated cSS in our cohort suggests active and widespread leptomeningeal CAA, causing repeated previous cSAH leading to cSS.

Although our study suggests that the risk of future sICH is highest in patients who fulfil the Boston criteria for probable CAA, it should be noted that eligibility for these criteria can change over time; indeed, four of nine patients with possible CAA or non-CAA who had subsequent ICH at follow-up then “converted” to probable CAA, but were still considered to be “non-probable CAA” for the purpose of the pooled analysis.

The pooled event rate of 19% per year for future ICH in patients with probable CAA is higher than the future risk of ischaemic stroke reported after ICH, which has been reported to be between 1.3 and 2.9% per year following deep ICH and 2.5–14.3% per year following lobar ICH [33]. Data on the risk of future ischaemic stroke following cSAH is scarce [21, 28], but our findings suggest that the risk of ICH is very likely to be greater than the risk of cerebral ischaemia. This finding is potentially clinically relevant when making decisions about antithrombotic drug use. Although data are not available on whether or how antithrombotic drug use might affect the future risk of sICH or ischaemia in patients with cSAH at risk of future vaso-occlusive events (e.g. those with atrial fibrillation or other vascular risk factors), our findings suggest that antithrombotic drugs might be best avoided after cSAH unless there is a very compelling clinical indication. Since the TFNE commonly associated with cSAH can closely mimic the typical symptoms of TIA (although they are more likely to be positive and of spreading onset) the diagnosis of cSAH is crucial in patients with suspected TIA. Although CT may detect cSAH, MRI might be preferable because it is sensitive to small areas of sulcal haemorrhage and can identify cortical superficial siderosis and CMBs, allowing a more confident diagnosis of cSAH related to CAA.

Our study has strengths. We used multiple overlapping methods of case ascertainment to identify all patients with cSAH presenting to our comprehensive stroke service. All visual rating of neuroimaging was undertaken by a trained observer blinded to outcome. Our systematic search followed established PRISMA guidelines and pooled all available studies using mixed effects Poisson regression to account for studies with no ICH events.

Our study also has limitations. Despite conducting a systematic search and pooling event rates in a meta-analysis, there were still few sICH events. Furthermore, as all studies are hospital-based there is potential for significant selection bias and detection bias (due to potentially selective follow up). We were unable to adjust for confounders such as hypertension, age, antithrombotic treatment, previous ICH, which can all contribute to sICH risk. Follow-up times were variable from study to study, but we did not have access to the data needed for a time-to-event analysis. We did not obtain individual patient data, and so were unable to explore whether imaging findings such as cSS, CMBs and leukoaraiosis modify the risk of ICH.

In summary, patients who present with an acute cSAH and suspected CAA have a substantial risk of symptomatic ICH (16% per patient-year overall). The future sICH risk might be highest in those with neuroimaging findings consistent with probable CAA. These estimates of sICH event rates should be of value to clinicans when counselling patients regarding their future risk, and emphasise the importance of offering treatments aiming to reduce this risk. We suggest that antithrombotic drugs (antiplatelet and anticoagulant agents) are probably best avoided wherever possible after proven cSAH, unless there is a compelling indication, since the future sICH risk is high, and likely to be increased by exposure to such drugs. In patients with cSAH and a strong indication for antithrombotic drugs (e.g. recent symptomatic ischaemic heart disease or peripheral vascular disease), the likely benefits on reducing vaso-occlusive events must be carefully weighed against the potential for increasing the sICH risk. In patients with cSAH it seems reasonable to reduce blood pressure, which is effective for the  long-term secondary prevention of ICH, including that attributed to probable CAA [34]. Although this data currently presents the best available evidence the small sample sizes and limited information on other risk factors remains a limitation; future prospective studies with individual patient data might help consolidate our observations.
